# Sustained Drug Free Remission of Lupus Panniculitis with Methotrexate: A Case Report From Nepal

**DOI:** 10.31729/jnma.3885

**Published:** 2018-12-31

**Authors:** Binit Vaidya, Rikesh Baral, Hritu Baral, Shweta Nakarmi

**Affiliations:** 1 Department of Rheumatology, National Center for Rheumatic Diseases, Ratopul, Kathmandu, Nepal

**Keywords:** *lupus*, *Methotrexate*, *Nepal*, *panniculitis*

## Abstract

Cutaneous manifestations are second most common presenting feature of lupus. Discoid lupus erythematosus is the most common variant amongst all; lupus panniculitis being described in only 2–5% of cases. Most cases of cutaneous lupus are associated with autoantibodies and either precede or follow the systemic manifestations of lupus. There is no proven treatment for cutaneous manifestations of lupus including lupus panniculitis. Available non-randomized studies show efficacy of hydroxychloroquine in most cases, whereas methotrexate and other immunosuppressant are used in relapsing cases.

We describe a case of lupus panniculitis presenting as isolated manifestation of lupus with negative autoantibody titers which responded well to methotrexate. We observed that lesions went into drug free remission in 1 year and did not recur on 1 year follow-up. There was no residual skin atrophy or scarring. Drug free remission in isolated cases of lupus panniculitis variant could be possible with timely intervention in the absence of autoantibodies.

## INTRODUCTION

Cutaneous manifestations are the second most common presenting feature of systemic lupus erythematosus (SLE).^1^ Around 80% develop skin disease during their course.^2^ Lupus panniculitis (LP) is an uncommon form of chronic cutaneous lupus erythematosus (CCLE).^3^ The diagnosis of LP is usually delayed and considered after non-resolution of skin lesions. Most of the patients with LP are ANA positive and either precede or follow the systemic manifestations of lupus.^4^ Current evidence suggests use of hydroxychloroquine; followed by methotrexate (MTX), azathioprine and mycophenolate in recalcitrant cases.^5^

We describe a case of young female with isolated lupus panniculitis who had sustained drug free remission with methotrexate.

## CASE REPORT

**Figure 1.**
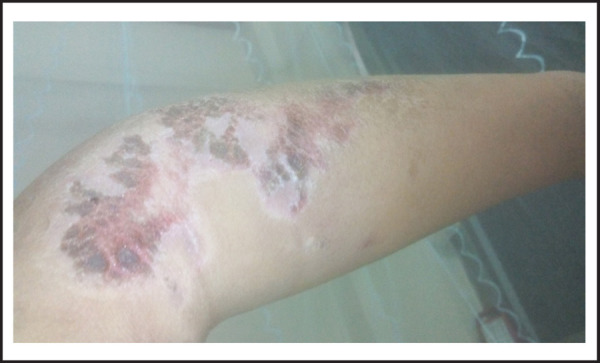
Presenting skin lesion.

**Figure 2.**
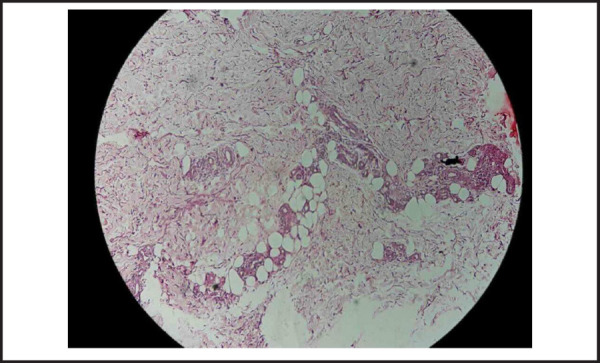
Low magnification.

**Figure 3.**
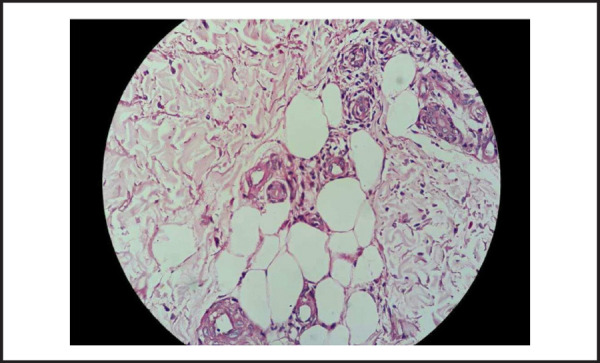
High magnification.

A 22-year-old female presented to us in August 2016 with painful, non-itchy, non-healing and progressively increasing skin lesion on her left arm region since 3 months (Fig 1). The lesions appeared as confluent areas of erythema and induration with superficial ulceration. She denied any history of previous trauma, surgery, insect bite or chemical exposure. There was no history of malar rash, hair loss, oral ulcers, Raynaud's phenomenon and joint pains. Examination revealed an ulcerated plaque with induration and erythema at the base with crusts and scabs on the surface and purulent discharge. Systemic examination was unremarkable. Laboratory evaluation revealed normal blood counts and biochemistry (Table 1). Anti-nuclear antibody (ANA) was positive in low titres (1:80 speckled) and extractable nuclear antigens (ENA) were absent; anti-cardiolipin IgG/IgM and lupus anti-coagulant were negative (Table 2); erythrocyte sedimentation rate (ESR) by Westergren's method was 42mm in 1^st^ hour and C-reactive protein (CRP) was 36.4 mg/L. Swab culture was sterile. Skin biopsy revealed atrophied epidermis with basal vacuolation and lymptocytic infilteration with septal panniculitis. There was no evidence of giant cells, granulomas or necrosis. A diagnosis of lupus panniculitis was made (Fig 2 and 3).

**Table 1:** Baseline parameters.

	BASELINE	3 MONTHS	1YEAR
HEMOGLOBIN (gm/dl)	12.0	11.4	11.9
WHITE BLOOD COUNT (per mm^3^)	8800	6700	5600
PLATELETS (per mm^3^)	155000	195000	179000
ERYTHROCYTE SEDIMENTATION RATE (ESR) mm at 1^st^ hour	42	40	20
CRP TITRE (mg/L)	36.4	3.1	1.2

**Table 2:** Results of immunological tests.

PARAMETER	RESULT	REFERENCE
ANA BY IFA	1:80 SPECKLED	<1:40
DSDNA	35U/ML	(<25)
ANTICARDIOLIPIN IGG/IGM	NEGATIVE	
LUPUS ANTICOAGULANT	NEGATIVE	
U1RNP	NEGATIVE	
ANTI SCL-70	NEGATIVE	
ANTI SMITH AB	NEGATIVE	
ANTI CENP AB	NEGATIVE	
ANTI RIBOSOMAL P PROTEIN	NEGATIVE	
ANTI RO 60	NEGATIVE	
ANTI RO 52	NEGATIVE	
ANTI LA AB	NEGATIVE	
ANTI PM-S	NEGATIVE	
ANTI JO-1	NEGATIVE	
ANTI MI-2	NEGATIVE	

*Confluent areas of erythema and induration with superficial ulceration on left arm of the patient at presentation.*

*Low power field showing areas of lymphocytic infiltration and collagen deposition.*

**Figure 4.**
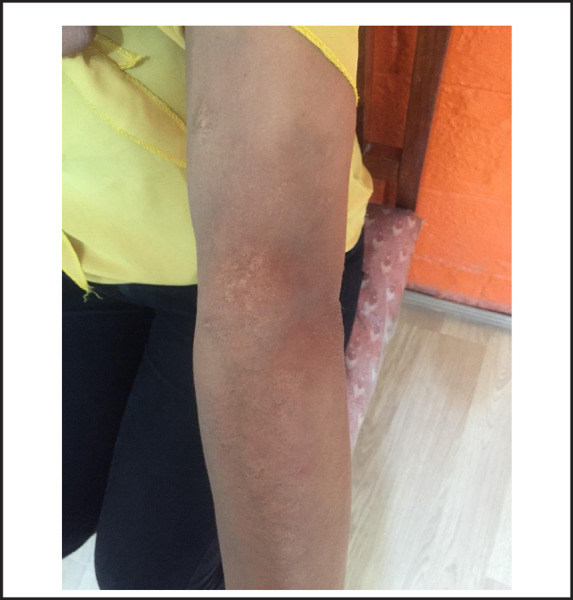
Follow-up at 2years.

She was started on prednisolone 20mg per day tapered by 5 mg every 2 weeks and hydroxychloroquine (HCQ) 200mg twice a day, along with a course of Flucoxacillin and topical mupirocin for 7 days for secondary bacterial infection. The lesion responded to steroids and significantly reduced in size over next 3 weeks. However, it again began to ulcerate when prednisolone was tapered to 5mg per day. Therapy was escalated to prednisolone 20mg (again tapered in same regimen) with the addition of methotrexate (MTX) 15mg once a week along with folic acid. HCQ was maintained throughout the period. The lesion gradually reduced in size and ulceration healed without recurrence over next 3 months. Prednisolone was maintained at 5mg every week for 3 months and then discontinued. She was observed on MTX and HCQ therapy for next 6 months. Her follow-up ESR and CRP were normal after initial 3months of MTX addition. After 6 months of steroid free remission, her MTX was gradually reduced at 5mg every 3months and after 9 month (i.e 1 year of treatment), HCQ was also stopped. She did not show any sign of relapse in the following year. There was post-inflammatory hyperpigmentation with no scarring or atrophy of overlying skin (Figure 4).

*High power field showing concentric areas of collagen deposition with perivascular and fat tissue lymptocytic infiltration*

*The patient's skin lesion improved with mild post-inflammatory hyperpigmentation with no scarring or atrophy of overlying skin at 2years of treatment.*

## DISCUSSION

Skin manifestations are the second most common presenting feature of lupus with three different subtypes: acute, subacute and chronic. Acute cutaneous lupus erythematosus constitutes<10% of cases whereas subacute forms account for around 20% cases. These acute and subacute form present with photosensitive rashes and heal without scarring. Majority of remaining cases are discoid lupus, presenting as erythematous, annular plaques with central hyperkeratosis healing with scarring and atrophy. Lupus panniculitis, also known as lupus profundus, is a rare variant occurring in 2 to 5% of SLE cases and is usually associated with systemic manifestations of lupus.^6^ The approximate risk of conversion of CCLE to full lupus is estimated around 12%.^3^ There are reports of lupus panniculitis as an initial manifestation of SLE, where patient developed full systemic symptoms after 2 years of panniculitis onset.^7,8^ Approximately half of patients with lupus panniculitis show some evidence of systemic disease. Our patient presented only with isolated unilateral skin lesion and did not show any systemic features during 2 years follow-up period.

LP usually presents as indurated, painful plaques or nodules with or without overlying cutaneous changes and lipoatrophy. Sites commonly affected are scalp, face, upper arms, chest (particularly breasts), lower back, flank, upper thighs, or buttocks. Ulceration and/or calcification may develop at the affected site. Characteristic histological findings are perivascular infiltrates of mononuclear cells plus panniculitis, manifested as hyaline fat necrosis with mononuclear cell infiltration and lymphocytic vasculitis.^9^

Studies have reported varying association of cutaneous lupus with ANA positivity and few described associations of anti-Ro antibodies with subacute and chronic forms of lupus, specially DLE.^4^ Our patient had low titer ANA with negative testing for all ENA. In the absence of systemic features and serological markers, the diagnosis was only clinched by biopsy.

Treatment of LP is quite difficult in absence of specific guidelines. Topical and intralesional corticosteroid is considered as initial management followed by anti-malarial therapy. A subset of patients who fail to respond to these therapies can be treated with MTX, MMF, Dapsone, Thalidomide, IVIG, Rituximab.^5^ MTX has found to be beneficial in the treatment of refractory CCLE.^10^ There are no randomized trials for therapeutic agent of choice for cutaneous lupus, especially LP. In few reports, patients responded well to HCQ^7^ and cyclophosphamide^8^ with tapering dosage of GC.^7^

Our patient also did not initially respond to HCQ but responded to MTX and systemic corticosteroids. Another unusual experience from this patient was the successful tapering of DMARDs after 1year of treatment and healing without scarring. There are no reports of sustained drug free remission of lupus panniculitis reported in literature.

This might suggest a possible drug free remission of isolated lupus panniculitis if treated early with DMARDs. On the other hand, it might be a milder form of self-limiting disease as suggested by absence of significant autoantibody profile and lack of systemic symptoms throughout the course of disease.

## References

[1] Cervera R, Khamashta MA, Font J, Sebastiani GD, Gil A, Lavilla P (1993). Systemic lupus erythematosus: Clinical and immunologic pattern of disease expression in a cohort of 1000 patients. Medicine..

[2] Cojocaru M, Cojocaru IM, Silosi I, Vrabie CD. (2011). Manifestations of Systemic Lupus Erythematosus. Maedica..

[3] Grönhagen CM, Fored CM, Granath F, Nyberg F. (2011). Cutaneous lupus erythematosus and the association with systemic lupus erythematosus: a population-based cohort of 1088 patients in Sweden. Br J Dermatol..

[4] Tebbe B, Mansmann U, Wollina U, Auer-Grumbach P, Licht-Mbalyohere A, Arensmeier M (1997). Markers in cutaneous lupus erythematosus indicating systemic involvement. A multicenter study on 296 patients. Acta Derm Venereol..

[5] Winkelmann RR, Kim GK, Del Rosso JQ. (2013). Treatment of Cutaneous Lupus Erythematosus: Review and Assessment of Treatment Benefits Based on Oxford Centre for Evidence-based Medicine Criteria. J Clin Aesthet Dermatol..

[6] Fraga J, Garcia-Diez A. (2008). Lupus erythematosus panniculitis. Dermatol Clin..

[7] Patel R M, Marfatia YS. (2010). Lupus panniculitis as an initial manifestation of systemic lupus erythematosus. Indian J Dermatol..

[8] Zhao Y-K, Wang F, Chen W-N, Xu R, Wang Z, Jiang Y-W (2016). Lupus Panniculitis as an Initial Manifestation of Systemic Lupus Erythematosus: A Case Report. Medicine (Baltimore)..

[9] LeBlanc RE, Tavallaee M, Kim YH, Kim J. (2016). Useful parameters for distinguishing subcutaneous panniculitis-like T-cell lymphoma from lupus erythematosus panniculitis. Am J Surg Pathol..

[10] Boehm IB, Boehm GA, Bauer R. (1998). Management of cutaneous lupus erythematosus with low-dose methotrexate: indication for modulation of inflammatory mechanisms. Rheumatol Int..

